# Normal Residual Lymphoid Cell Populations in Blood as Surrogate Biomarker of the Leukemia Cell Kinetics in CLL BinetA/Rai 0

**DOI:** 10.3390/cancers17030347

**Published:** 2025-01-21

**Authors:** Fernando Solano, Ignacio Criado, Nahir Moreno, Carlos Gomez-Gonzalez, Ana Lerma-Verdejo, Cristina Teodosio, María Dolores Martinez-Moya, Iryna Luts, Teresa Contreras, Guillermo Oliva-Ariza, Blanca Fuentes Herrero, Jose Manuel Serrano-Lozano, Julia Almeida, Alberto Orfao

**Affiliations:** 1 Hematology Service, Hospital General Universitario Nuestra Señora del Prado, 45600 Talavera de la Reina, Spain; fsolano@sescam.jccm.es (F.S.); nahirm@sescam.jccm.es (N.M.); cg@sescam.jccm.es (C.G.-G.); alerma@sescam.jccm.es (A.L.-V.); mmamo@sescam.jccm.es (M.D.M.-M.); iluts@sescam.jccm.es (I.L.); 2 Translational and Clinical Research Program, Cancer Research Center (IBMCC, CSIC—University of Salamanca), 37007 Salamanca, Spain; ignaciocriado@usal.es (I.C.); cristina.teodosio@usal.es (C.T.); goliva@usal.es (G.O.-A.); blancafh@usal.es (B.F.H.); jserranolozano@usal.es (J.M.S.-L.); jalmeida@usal.es (J.A.); 3 Cytometry Service, NUCLEUS, Department of Medicine, University of Salamanca (Universidad de Salamanca), 37007 Salamanca, Spain; 4 Institute of Biomedical Research of Salamanca (IBSAL), 37007 Salamanca, Spain; 5 Biomedical Research Networking Centre Consortium of Oncology (CIBERONC), Instituto de Salud Carlos III, 28029 Madrid, Spain; 6 Biochemistry Service, University Hospital of Salamanca, 37007 Salamanca, Spain; tcontrerass@saludcastillayleon.es

**Keywords:** chronic lymphocytic leukemia, flow cytometry, lymphocyte doubling time, immunity, time to first therapy

## Abstract

Background/Objectives: Despite the current international prognostic index for chronic lymphocytic leukemia (CLL) being widely accepted and broadly used, it does not consider the kinetics of the B-cell clone over time. Here, we investigated the potential association between distinct features of leukemic cells and other immune cells in blood and the kinetics of clonal B-cells in CLL stage Binet A/Rai 0 (A/0) patients; Methods: Based on the leukemia cell kinetics, 69 CLL A/0 cases followed for a median of 105 months were classified as carrying stable (*n* = 53) vs. rapidly increasing in size (*n* = 16) CLL clones; Results: Patients with increasing CLL clones had a significantly higher risk of disease progression and shortened time to first therapy vs. those carrying stable B-cell clones (*p* ≤ 0.001). Strikingly, the distribution of various immune-cell populations in blood at diagnosis also differed significantly between the two groups, with lower Tαβ CD4^+^CD8^lo^ cell counts (*p* = 0.03), a greater switched/unswitched memory B-cell ratio (*p* = 0.01), and higher plasma cell counts (*p* = 0.05) in CLL with increasing vs. stable clones. Multivariate analysis revealed that the number of circulating clonal B-cells (≥15 × 10^9^/L) and Tαβ CD4^+^CD8^lo^ cells (≤35 cells/µL), together with an *IGHV* unmutated gene status at diagnosis, were independent predictors of an increasing CLL clone; Conclusions: Altogether, these data suggest that the expansion of the CLL clone in stage A/0 patients may depend on both the intrinsic characteristics of CLL cells and the surrounding immune microenvironment.

## Simple Summary

The current prognostic index for chronic lymphocytic leukemia does not account for the dynamic changes in the B-cell clone over time. This study aims to investigate the association between the tumor microenvironment and the kinetics of clonal B-cells in early-stage CLL patients. By categorizing patients based on the rate of clonal B-cell increase, we identified significant differences in immune cell profiles and clinical outcomes. Patients with rapidly increasing clones exhibited poorer prognosis and shorter time to treatment, but notably, lower Tαβ CD4^+^CD8^lo^ cell counts, altered B-cell subsets, and higher plasma cell counts were associated with highly proliferative clones. Multivariate analysis confirmed that the number of clonal B-cells, Tαβ CD4^+^CD8^lo^ cells, and the *IGHV* mutational status were independent predictors of clonal expansion. These findings suggest that the interplay between CLL cells and the immune microenvironment might play a relevant role in disease progression, potentially leading to the development of novel prognostic markers.

## 1. Introduction

Chronic lymphocytic leukemia (CLL) displays a very heterogeneous clinical course and variable outcome. Thus, while some patients rapidly progress to advanced stages, others remain stable for long periods of time, in the absence of symptomatic disease and no need for therapy [1,2]. In the last decades, a major effort has been put into identifying (biological and clinical) biomarkers that might help predict the prognosis and outcome of CLL on an individual patient basis. Such effort has led to the CLL International Prognostic Index (CLL-IPI), which helps predict patient outcome [e.g., 5-year overall survival (OS)] based on five well-established prognostic markers: the *IGHV* and *TP53* mutational status of CLL cells, the patient’s age and clinical stage, and β2-microglobulin (β2m) serum levels at diagnosis [3,4].

Despite the CLL-IPI including some laboratory features such as the *IGHV* mutational status and *TP53* alterations, no specific biomarker directly reflecting the kinetics of the CLL clone, independent of the tumor mass, such as the lymphocyte doubling time (LDT), is considered [3,5,6,7]. In fact, LDT has long been identified as a relevant prognostic marker for stratification of CLL stage Binet A/Rai 0 (A/0) [5,8]. More recent reports further suggest that combined assessment of the LDT and the CLL-IPI (or a subset of the CLL-IPI variables) might improve the prediction of the outcome of CLL patients [6,9,10]. However, assessment of the LDT in individual patients requires a follow-up of up to 12 months to discriminate between cases with low vs. fast tumor growth, as reflected by the period required, per definition, to assess the time needed to double the number of peripheral blood (PB) lymphocytes [5]. This, together with the lack of an alternative surrogate biomarker that would reflect the kinetics of the leukemia B-cell clone already at diagnosis, has hampered its generalized use in routine clinical practice.

Increased evidence indicates that in addition to the leukemia cell intrinsic features and the overall tumor load at diagnosis, the tumor-cell microenvironment, particularly the alterations in the innate and adaptive immune cells, may also play a critical role in the biology of CLL [11,12,13]. Thus, it is currently well-established that B-cell receptor (BCR)-mediated signaling is critical for the activation of CLL cells, which produce cytokines that can be sensed by themselves and by bystander cells [14,15]. This also results in the activation of surrounding immune cells, which in turn may produce cytokines that promote the survival of leukemia cells [11]. Notable examples of this crosstalk include the activation of the CD40-CD40L axis in T-cells, leading to the production of IL-4 and IL-10, upregulation of SOCS5 preventing STAT6 activation, and the polarization of macrophages towards an M2 phenotype, among other effects [11,16,17,18]. Such crosstalk between CLL cells and bystander immune cells might vary among individual patients, leading to abnormal patterns of distribution of normal residual B- and T-cell populations in different tissue compartments, including blood [19,20,21,22,23,24]. In this regard, recent data support a close association between the kinetics of monoclonal B-cell lymphocytosis (MBL) cells and other blood-circulating T-cell populations, such as Tαβ CD4^+^CD8^lo^ and Tαβ CD8^+^CD20^+^ cells [25,26,27,28].

In this exploratory study, we prospectively evaluated the distribution and kinetics of clonal B-cells and normal lymphocyte populations in the blood of a real-world cohort of newly diagnosed CLL stage A/0 patients, aiming at identifying (already at diagnosis) surrogate cellular biomarkers of tumor cell kinetics, which might help to identify patients at greater risk of disease progression with a shortened time to first therapy (TFT).

## 2. Materials and Methods

Patients and controls: A total of 69 patients diagnosed with CLL [32 men (46%) and 37 women (54%)]; median age at diagnosis of 73 years (range: 49–87)] were sequentially recruited between January 2011 and November 2017 and followed every 6 months (or until disease progression requiring therapy) for a median (range) period of 105 months (13–137 months) at the Hematology Service of Nuestra Señora del Prado University Hospital (Talavera de la Reina, Spain). Diagnosis was made according to the WHO [29] and the International Workshop on CLL (iwCLL) criteria [1]. Only those patients diagnosed with CLL A/0 who had not received therapy for CLL (or any other tumor), including the use of immunomodulatory agents (e.g., corticosteroids), were enrolled in this study after they had given their written informed consent to participate. The study was approved by the Ethics Committee of Nuestra Señora del Prado University Hospital. Every patient included in the study was categorized into either the “stable” or “increasing” CLL clone groups according to the kinetics of the leukemia B-cell clone size in PB within the first 12 months after diagnosis, based on the following criteria: (1) duplication of the absolute number of blood-circulating CLL cells; and/or (2) an increase of ≥5 × 10^9^ clonal B-cells/L over the baseline diagnostic CLL cell counts in blood in the first 12 months after diagnosis. Clinical progression was defined according to the National Cancer Institute Workgroup (NCI-WG) and the iwCLL guidelines, as follows: progressive bone marrow (BM) failure, massive or progressive lymph nodes and/or splenomegaly, LDT < 6 months, autoimmune complications, extranodal (symptomatic) involvement, and emergence of disease-related B-symptoms, such as night sweats, fever, and unintentional weight loss of ≥10% within 6 months [1]. Importantly, all patients in this study who demonstrated clinical progression required therapeutic intervention (i.e., need for treatment).

A total of 246 sex- and age-matched healthy donors (HD) from the general population, with a median (range) age of 70 years (44–99 years), defined as having no active disease or ongoing infection at the time of sampling, no previous history of hematologic malignancies, MBL, or monoclonal T-cell expansion of undetermined significance (TCUS), in the absence of any immunomodulatory treatment, were used as controls.

Immunoglobulin (Ig), β2-microglobulin (β2m), and lactate dehydrogenase (LDH) plasma levels: Soluble plasma levels of IgM and IgG were measured by conventional nephelometry (Dimension Vista; Siemens Healthcare, Erlanger, Germany), while IgA was determined by the SPAplus^®^ turbidimetric system (Binding Site, Birmingham, UK). The titers of serum CMV-specific IgM and IgG antibodies were assessed with the Alinity ci CMV IgM and Alinity ci CMV IgG assay kits (Abbott, Green Oaks, IL, USA). In parallel, β2m and LDH serum levels were measured at every study time-point by conventional biochemical assays.

Immunophenotypic studies: Between 1 and 2 mL of EDTA-anticoagulated PB were lysed and stained within 24 h after sample collection, following the EuroFlow BulkLysis protocol described at www.EuroFlow.org. The specific reagents and protocols used are detailed in Table S1A,B. Subsequently, ≥2 × 10^6^ stained leukocytes/tube were measured in an 8-color FACSCanto II flow cytometer –Becton/Dickinson Biosciences (BD), San Jose, CA–. Instrument setup, calibration, and daily quality control were performed according to well-established protocols [30]. For data analysis, the Infinicyt^TM^ software V.2.1.0 (BD Biosciences-Cytognos, Salamanca, Spain) was used. Identification of CLL cells, as well as of other coexisting normal residual B-cell and T-cell subsets, was performed in individual PB samples, based on their unique immunophenotypic profiles in blood as detailed in Table S1C and illustrated in Figure S1 [31,32,33]. In every case, the absolute counts of CLL cells and other normal lymphocyte populations were calculated using a dual platform assay based on the white blood cell (WBC) count obtained in a DXH 800 hematological cell counter (Beckman/Coulter, Brea, CA, USA) and the flow cytometric-based frequency (percentage from total leukocytes) values of each individual cell subset (from WBC) as follows: absolute cell count (cells × 10^9^/L) = (% of cell population × WBC)/100 [34].

Interphase fluorescence in situ hybridization (iFISH) and molecular studies: CLL-associated cytogenetic alterations –i.e., del(13q14)(D13S25), trisomy 12, del(11q)(ATM), and del(17p)(TP53)– were investigated in whole blood using previously described [35] conventional iFISH procedures, based on the set of probes detailed in Table S2.

The IGHV mutational status of clonal B-cells was assessed on genomic DNA (gDNA) obtained from whole PB by sequencing the unique VDJ rearrangement of individual patients, followed by direct comparison of the sequences obtained to germline sequences in the IMGT database (https://www.imgt.org/IMGT_vquest (accessed on 16 January 2025)), based on previously described standard operating procedures (SOPs) [36,37]. Sequences containing > 2% deviation from the germline sequence were considered to be somatically mutated (M), while those with ≤2% deviation were classified as unmutated (UM), following well-established consensus criteria [38].

Statistical analyses: Conventional descriptive and comparative statistics for independent (Mann–Whitney U test) and paired (Wilcoxon test) continuous variables and for categorical variables (Chi-square and Fisher’s exact tests) were applied. Time-dependent survival curves were plotted according to the Kaplan–Meier method, and differences between curves were assessed with the (one-sided) log-rank test. For multivariate analysis, both the binary logistic and Cox regression models were used to identify those variables independently associated with the risk of duplication and/or growth of the CLL clone and TFT. For both Kaplan–Meier and logistic regression analyses, all continuous variables were transformed into Boolean variables, based on cut-off values determined by receiver operating curve (ROC) analysis. All statistical analyses were performed with SPSS 23.0 (SPSS-IBM, Armonk, NY, USA), and graphical plots were drawn with GraphPad Prism V.8 (GraphPad, San Jose, CA, USA). *p*-values ≤0.05 and ≤0.1 were associated with statistical significance and a trend toward statistical significance, respectively.

## 3. Results

### 3.1. Leukemia Cell Kinetic Profiles in CLL Stage A/0 and Disease Features at Diagnosis vs. Follow-Up

A total of 69 newly diagnosed CLL stage A/0 patients (median age of 73 y; range: 49–87 years) were studied. None of these patients showed lymphadenopathies, anemia, and/or thrombocytopenia at diagnosis; their median lymphocyte count at diagnosis was of 15 × 10^9^ cells/L (9.3–69 × 10^9^ cells/L) (Table S3). After a median follow-up of 105 months, 14/69 (20%) patients had shown disease progression and required treatment for CLL.

Based on the kinetics of the B-cell clones, CLL A/0 patients were divided into cases with stable (*n* = 53; 77%) vs. increasing (*n* = 16; 23%) CLL clones (Table S4), following the above-defined criteria. Overall, significantly higher WBC, lymphocyte, and CLL cell counts were found in the blood of CLL patients carrying increasing vs. stable clones both at diagnosis (*p* ≤ 0.001) and at the last follow-up time point (*p* ≤ 0.004) (Table S4). Interestingly, 8/16 (50%) patients with increasing CLL clones required therapy, whereas only 6/53 (11%) patients with stable CLL clones needed treatment (*p* = 0.02; Table S4). Furthermore, a significantly higher number of unmutated *IGHV* clones was observed among CLL stage A/0 cases with increasing vs. stable clones (50% vs. 8.7%, respectively, *p* = 0.001; Table S4). In Table S5, detailed features of the individual *IGHV* genes used by the CLL clones and their relationship to the kinetics of the corresponding clonal B-cell populations are shown. As expected, significantly higher WBC (*p* < 0.001), lymphocyte (*p* < 0.001), and CLL cell counts (*p* < 0.001) were found at the last follow-up study vs. at diagnosis among patients with increasing, but not those with stable CLL clones (Table S4).

Given the well-documented hypogammaglobulinemia in CLL patients and prior studies demonstrating hypogammaglobulinemia with persistently stable antibody levels against latent viruses like CMV and EBV [23], we investigated the levels of CMV-specific soluble IgM and IgG antibodies, which were found to be similar between CLL patients with increasing vs. stable clones (Table S6).

### 3.2. Distribution of Normal Residual B- and T-Cell Populations in Blood According to the CLL Clone Kinetics Profile

Normal residual B lymphocytes were significantly reduced at diagnosis in CLL A/0 patients compared to sex- and age-matched HD (median: 82 vs. 160 B-cells/µL, respectively: *p* ≤ 0.001). Such a decrease was mostly at the expense of lower pre-germinal center (GC) B lymphocyte counts [i.e., immature B-cells, naïve B lymphocytes (*p* ≤ 0.001)], and, to a lesser extent, also sIgM^+^ unswitched-memory B-cells (MBC) (*p* ≤ 0.001) (Table S7).

Once we compared the distribution of the different B-cell subsets in the blood of CLL patients with increasing vs. stable CLL clones, significantly higher PC counts were observed in blood among the former cases, both at diagnosis (*p* = 0.05) and at the last follow-up time-point (*p* = 0.03) (Table 1, Tables S8 and S9). In addition, the ratio between switched (sIgM^−^) and unswitched (sIgM^+^) MBC (sIgM^−^/sIgM^+^ MBC ratio) was higher in CLL cases with increasing vs. stable cell clones (*p* = 0.04), despite in both patient groups this MBC ratio was significantly increased (*p* ≤ 0.001) compared to age-matched HD (Table 1). Subsequently, the absolute number of normal residual B-cells decreased significantly during follow-up among cases carrying stable CLL clones (*p* = 0.001), due to progressively lower counts of naïve B-cells (*p* = 0.005) and switched (sIgM^−^) MBC (*p* ≤ 0.001). In contrast, CLL A/0 cases with increasing CLL clones only showed decreasing switched (sIgM^−^) MBC counts during follow-up (*p* = 0.02) (Table 1). A more detailed comparison of the distribution of the B- and T-cell populations analyzed by age in the CLL patients vs. HD is presented in Tables S8 and S9.

### 3.3. Identification at Diagnosis of Surrogate Biomarkers of the CLL Clone Kinetics

**Figure 1:**
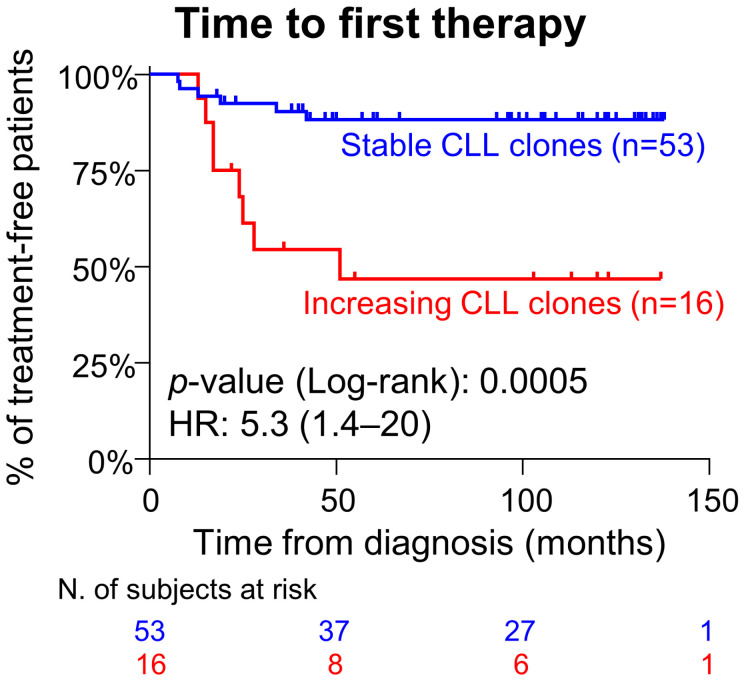
Impact of CLL stage A/0 clone kinetics on patient progression-free survival. Results expressed as hazard ratio (95% confidence interval). Blue and red lines represent CLL patients with stable and increasing CLL clones, respectively. Vertical lines depict censored cases. Abbreviations (alphabetical order): CLL, chronic lymphocytic leukemia; HR, hazard ratio; N., number; 95%CI, 95% confidence interval.

Once we compared CLL patients with increasing vs. stable B-cell clones, the former cases showed a significantly shorter TFT due to disease progression [hazard ratio (95%CI): 5.3 (1.4–20); *p* = 0.0005; Figure 1].

**Figure 2:**
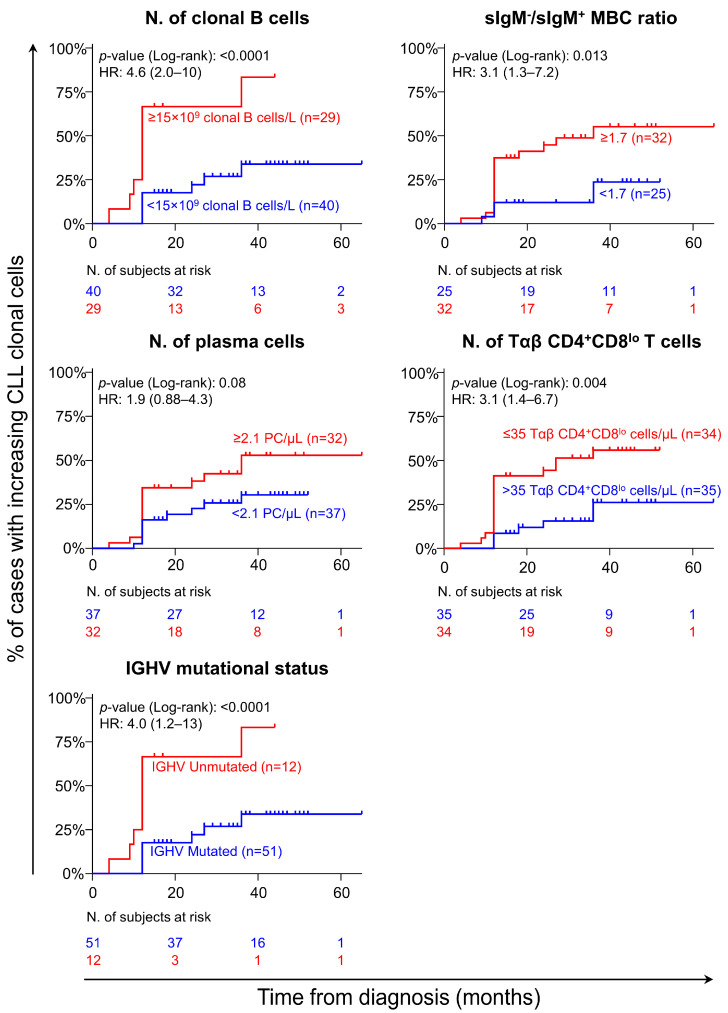
Association between the absolute number of blood-circulating normal residual B- and T-cell populations and the kinetics of clonal cells in CLL A/0 patients. Plots represent the frequency of cases that significantly increased their clone size over time. Vertical lines depict censored cases. Abbreviations (alphabetical order): FU, follow-up; *IGHV*, immunoglobulin heavy chain gene; MBC, memory B cell.

Since a follow-up period of at least 6–12 months was required to evaluate the kinetics of the CLL clone in individual patients, we further investigated whether there were biomarkers already measurable at diagnosis that may help identify CLL patients carrying increasing clones. Univariate analysis revealed that the presence of ≥15 × 10^9^/L clonal B-cells in blood and their *IGHV* mutational status (UM), together with a greater (≥1.7) sIgM^−^/sIgM^+^ MBC ratio, higher PC counts (≥2.1 cells/µL), and lower Tαβ CD4^+^CD8^lo^ cell numbers (≤35 cells/µL) in blood, were all associated with an increasing CLL clone profile (Figure 2). Multivariate analysis revealed that from these variables, only the presence of ≥15×10^9^/L clonal B-cells, ≤35 Tαβ CD4^+^CD8^lo^ cells/µL, and an unmutated *IGHV* gene status were independent variables to predict CLL with rapidly growing B-cell clones (Table 2). In line with these findings, a greater number of clonal B-cells (≥15 × 10^9^/L) and total PC counts (≥3.3 cells/µL), the presence of del(17p) (*TP53*), and an unmutated *IGHV* gene status were all associated with a shorter TFT among CLL A/0 patients (Figure S2). Interestingly, the proportion of clonal B-cells carrying del17p (*TP53*) among the three cases found to have this alteration (all of them with stable CLL clones) was highly variable: 7%, 58%, and 98%, suggesting that its predictive value for TFT might be independent from the tumor cell kinetics. When considering disease progression (i.e., time to first treatment), only the absolute number of ≥15 × 10^9^/L clonal B-cells and an unmutated *IGHV* status, along with ≥3.1 cells/µL PCs, were significantly associated with TFT in the univariate analysis, the number of clonal B-cells and an unmutated *IGHV* status retaining their significance in the multivariate and Cox regression analyses (Table S10).

## 4. Discussion

For decades, the LDT has been identified as a reliable biomarker of disease progression with a strong impact on patient outcomes [5]. However, this parameter has not been included in the most widely used risk stratification models for CLL, such as the CLL-IPI, because at least a period of 6- to 12-months is necessary to assess this parameter, and therefore it is not readily available at diagnosis.

Here, we investigated the potential value of parameters already available at diagnosis that may act as surrogate biomarkers of the kinetics of leukemia cells for the discrimination between CLL A/0 patients with stable vs. increasing leukemia cell clones and, therefore, to identify those at higher risk of and in need of therapy associated with disease progression. From a clinical perspective, the identification of ≥15 × 10^9^/L clonal B-cells at diagnosis is a straightforward and reliable parameter that can be easily assessed using flow cytometry. Interestingly, those patients with fast-growing clones showed similar LDH and β2m serum levels and overlapping cytogenetics at diagnosis, indicating they might be surrogate markers of the tumor burden but not of the CLL clone proliferation rate. In contrast, overrepresentation of poorer prognosis CLL with unmutated *IGHV* [38] among CLL A/0 patients with fast-growing vs. stable clones was observed. It is important to note that the present study included a limited number of cases exhibiting cytogenetic alterations known to be associated with poor clinical outcomes, particularly those carrying del17p (*TP53*) and/or del11q(*ATM*) [39]. This limited sample size may have obscured the true prognostic significance of these alterations (already present at the CLL A/0 stage) in predicting the dynamics of clonal B-cell populations. In this regard, to further investigate the impact of these variables on clonal B-cell dynamics, a larger multicenter study with an increased number of participants is warranted. Despite the prognostic impact of the kinetics profile of circulating CLL cells as a surrogate biomarker for TFT in CLL A/0 patients, this parameter is not readily available at diagnosis [4,5]. Because of this, here we focused on the identification of potential surrogate markers of the CLL clone kinetics that could be directly assessed at diagnosis [40,41].

The specific biological mechanisms that govern the dynamics of cancer cell populations, as well as the potentially relevant biomarkers associated with the kinetics of the CLL clone and patient outcome, remain largely unknown. However, accumulated evidence indicates that the crosstalk between CLL cells and their microenvironment due to direct cell–cell interactions, together with the effects of cytokines and chemokines secreted and sensed by both the tumor cells and the bystander immune cells, might trigger the activation of CLL cells and promote their expansion [11,42,43]. In this regard, previous studies have recurrently reported an altered distribution and functionality of specific populations of blood-circulating B- and T-cells at the earliest stages of the disease, from low-count (MBL^lo^) to high-count MBL (MBL^hi^) [24,25,44], in addition to CLL [22,24]. Despite this, the potential association between the kinetics of the tumor cell population and the distribution of the main B- and T-lymphocyte subsets in blood has not been previously investigated. As expected, our results showed a significant decrease (vs. HD) of normal residual B-cells in the blood of CLL A/0 patients, already at diagnosis. Such a decrease was mostly at the expense of pre-GC B-cells, and it involved patients with both stable and increasing CLL clones. These observations are in line with previous results from our group about the existence of a decreased B-cell production in CLL, which is already detectable in blood at the pre-leukemia stages of MBL^lo^ and MBL^hi^ [24]. Altogether, these findings suggest that the production of pre-GC B-cells in these subjects might be affected by the progressively higher invasion of the BM B-cell niches by CLL cells from MBL^lo^ to MBL^hi^ and CLL [24]. This might translate into a significantly restricted naïve B-cell repertoire against potential infectious agents, responsible, at least in part, for the impaired immune response and greater frequency and severity of infections observed in CLL as well as in MBL [23]. In contrast with pre-GC B-cells, the number of (sIgM^−^) switched MBC in blood was significantly increased in CLL patients at diagnosis (vs. HD), both in cases with stable and (more prominently) with increasing CLL clones. Of note, those cases presenting with increasing CLL clones also showed a deeper decrease in sIgM^+^ MBC counts in blood, which translated into a significantly higher sIgM^−^/sIgM^+^ MBC ratio among CLL patients with increasing vs. stable clones. These findings may reflect a reduced production of sIgM^+^ MBC derived from naïve cells that encountered their cognate antigen in primary immune responses, due to progressive depletion of the repertoire available to recognize new antigens, particularly evident here for those patients carrying increasing (vs. stable) CLL clones. Of note, (sIgM^−^) switched MBC counts also tended to decrease during follow-up, but at a lower rate than that of pre-GC and unswitched MBC, probably fueled by the reactivation of pre-existing MBC against widely spread pathogens, such as CMV and EBV [23]. In line with this hypothesis, the number of blood-circulating PC increased during follow-up in both patients with stable and increasing CLL clones, with similar CMV-specific IgM and IgG antibody titers in plasma in both groups of CLL A/0 patients, in line with previous observations [23].

In addition to the decreased pre-GC B-cell counts, previous reports have recurrently described increased numbers of dysfunctional (e.g., exhausted) T-cells in CLL, characterized by an increased expression of inhibitory receptors and decreased cytokine production and proliferative capacity [20,22,45,46,47]. Consistent with previously reported data, CLL A/0 patients showed significantly increased counts of total T-cells and their major populations in blood, compared to age- and sex-matched HD, in both the stable and the increasing CLL clone groups. Moreover, a statistically significant increase in all major T-cell subsets was observed during follow-up, independently of the CLL clone kinetics. Previous studies suggested that the overall increase in the number of circulating T-cells, and particularly of Tαβ CD4^−^CD8^+^ cells, might result from an underlying immune response to tackle the re-activation of host pathogens (e.g., CMV and EBV), in line with the persistently maintained anti-CMV specific IgM and IgG serum levels in a background of a progressively more severe hypogammaglobulinemia, as confirmed here for CLL A/0 with both stable and increasing leukemia cell clones [23]. However, when we compared the distribution of different T-cell subsets in both groups of CLL A/0, patients with fast-growing clones showed lower numbers of Tαβ CD4^+^CD8^lo^ cells compared to stable CLL A/0 cases.

Univariate analysis revealed that in addition to Tαβ CD4^+^CD8^lo^, both the sIgM^−^/sIgM^+^ MBC ratio and the PC counts in blood were all associated with the kinetics of the CLL clones. These findings suggest an increase in recently produced PC (counts) in blood due to an increased baseline B-cell response in parallel to progressively faster growth of the CLL clone; this might translate into a progressively more extensive invasion of specific B-cell and PC niches in the BM, with a potential inhibitory effect on PC homing, leading to increased PC counts in blood associated with hypogammaglobulinemia, a hallmark of CLL [22,45,48]. A deeper phenotypic and molecular analysis of circulating PC might shed light on the specific mechanisms that lead to the deterioration of the immune system in these patients. In line with the close association between increased PC and CLL counts, in the multivariate analysis only CLL and Tαβ CD4^+^CD8^lo^ cell counts, together with the *IGHV* mutational status, were independent predictors of the (stable vs. increasing) behavior of the clone in CLL A/0 patients. Of note, both the number of clonal B-cells and the *IGHV* mutational status assessed at diagnosis (but not the Tαβ CD4^+^CD8^lo^ cell counts) were biomarkers for predicting both the clonal population kinetics and TFT. Still, Tαβ CD4^+^CD8^lo^ cell counts at diagnosis would contribute to the identification of patients at risk of CLL cell growth, independently of the former two variables.

The underlying mechanisms that link T-cells and tumor growth are currently poorly understood. In this regard, it has been suggested that the crosstalk between B- and T-lymphocytes via the CD40-CD40L and the cytokines/chemokines released by both the tumor and T-cell compartments (e.g., CCL3, CCL4, CCL22, and IFN-γ, IL4, IL21, sCD40L, respectively) might activate signaling pathways that promote the proliferation and survival of CLL cells [12,16,42,49,50]. However, limited information is currently available regarding the precise physiopathologic role of Tαβ CD4^+^CD8^lo^ cells [51]. Despite this, many evidences indicate that this is a cytotoxic-like cell population with a presumably MALT-related origin [52,53]. According to their cytotoxic role, these cells show an effector memory or terminal effector cell phenotype, with frequent expression of CD56 and CD57 and a restricted TCR-Vβ repertoire, exerting their cytotoxic function through the release of cytolytic enzymes (e.g., granzyme B) following direct contact with the target cells, via an HLA class II-dependent and Fas/TRAIL-independent mechanism [54,55]. From the clinical point of view, Tαβ CD4^+^CD8^lo^ cells have been found to be expanded in response to CMV [56,57], as well as in different types of cancer, including CLL, which strongly supports its potential role in anti-tumoral immunosurveillance against HLA-II^+^ cells, like CLL cells [58,59,60,61]. Preclinical studies have demonstrated that this population might be expanded in the context of cytotoxic T CD8^+^-cell (CTL) impairment, as found previously in CLL [62,63,64]. Altogether, these findings suggest that the expansion of CD4^+^ CTL and/or Tαβ CD4^+^CD8^lo^ cell populations may represent a regulatory mechanism to control tumor growth via direct tumor cell killing, which might explain why the lower numbers of those cells in CLL emerged here could be used as a surrogate marker of the dynamics of clonal CLL cells. Despite all the data referred to above regarding the potential crucial role of T-cells and certain T-cell subsets in controlling tumor growth, whether the T-cell populations here found to be expanded in CLL are tumor- or CMV-specific T-cells, as well as their precise functional role and active vs. senescent/exhausted status, deserve further investigations in larger series of patients, to better understand the precise role of TCRαβ CD4^+^CD8^lo^ T-cells (and other adaptive and innate cells) in the tumor microenvironment and the kinetics of the CLL clone in blood.

## 5. Conclusions

In summary, here we confirm and extend on previous observations showing an altered distribution of blood circulating normal residual B- and T-cell populations in early-stage CLL A/0 patients compared to age-matched HD, with significantly different profiles in patients with stable vs. fast-growing leukemia cell clones. Most interestingly, the number of circulating Tαβ CD4^+^CD8^lo^ cells, together with the *IGHV* mutational status, were independent surrogate biomarkers of the CLL clone kinetics and may be used to identify at diagnosis CLL A/0 patients at higher risk of disease progression. Further prospective studies in larger series of patients in multicenter settings are needed to confirm our results and better understand the association here reported between Tαβ CD4^+^CD8^lo^ cells and the CLL cell kinetics.

## Figures and Tables

**Table 1:** Distribution of normal B- and T-cell populations in blood of CLL patients classified according to the kinetics of CLL clonal B-cells in blood.

	Sex- and Age-Matched HD	Stable CLL Clones (*n* = 53)		Increasing CLL Clones (*n* = 16)		*p*-Value
		Diagnosis	Last Follow-Up	Diagnosis	Last Follow-Up	
N. of normal B-cells/µL						
Total B-cells	160 (35–384)	83 *** (28–224)	56 (19–152)	75 *** (18–194)	74 (14–266)	0.001 ^c^
Immature	5.7 (<0.01–36)	2.7 ** (0.24–17)	1.7 (<0.01–12)	1.6 * (0.50–8.7)	2.6 (<0.01–18)	NS
Naive	79 (6.0–378)	14 *** (1.1–64)	8.8 (1.5–43)	9.9 *** (2.3–29)	9.1 (1.7–136)	0.005 ^c^
MBC	51 (8.0–185)	64 (17–205)	31 (11–90)	64 (4.2–158)	42 (3.1–132)	0.001 ^c^
MBC/Naive ratio	0.67 (0.04–4.5)	3.6 *** (0.74–38)	3.0 (0.51–44)	4.5 *** (1.6–16)	3.5 (0.31–37)	NS
sIgM^+^ MBC	26 (2.1–114)	11 ** (0.08–62)	9.6 (0.91–35)	5.7 * (1.2–84)	14 (0.89–64)	NS
sIgM^−^ MBC	29 (5.6–98)	45 *** (12–149)	19 (4.6–66)	62 *** (3.0–108)	28 (2.2–82)	≤0.02 ^c,d^
sIgM^−^/sIgM^+^ MBC ratio	1.2 (0.30–6.5)	1.6 * (0.49–40)	1.9 (0.40–16)	5.9 *** (0.74–18)	1.9 (0.22–4.9)	≤0.04 ^a,d^
PC	1.8 (<0.01–24)	1.5 (<0.01–12)	1.7 (0.01–20)	3.4 * (<0.01–19)	4.3 (0.01–36)	≤0.05 ^a,b^
N. of normal T-cells/µL						
Total T-cells	1129 (435–3068)	2689 *** (1335–6549)	2531 (1371–5597)	1991 *** (1170–7382)	2534 (1166–15,857)	0.008 ^d^
Tαβ CD4^+^CD8^−^ cells	620 (196–3224)	1319 *** (634–3193)	1415 (459–2679)	1160 *** (648–4140)	1517 (661–9038)	0.006 ^d^
Tαβ CD4^−^CD8^+^ cells	366 (13–1939)	850 *** (273–2556)	992 (267–2241)	608 *** (194–2474)	932 (184–5468)	0.02 ^d^
Tαβ CD4^+^CD8^−^/ CD4^−^CD8^+^ cell ratio	1.9 (0.31–43)	1.8 (0.41–4.4)	1.5 (0.40–4.3)	1.7 (0.60–3.5)	1.7 (0.95–5.2)	NS
Tαβ CD4^+^CD8^+^ cells	7.4 (0.70–94)	49 *** (6.8–203)	30 (2.0–139)	46 (4.7–213)	47 (1.7–258)	NS
Tαβ CD4^+^CD8^lo^ cells	15 (2.4–436)	45 *** (6.2–560)	40 (6.4–435)	25 (5.4–199)	36 (6.8–303)	0.03 ^a^
Tαβ CD4^−^CD8^−^ cells	11 (2.0–39)	143 *** (30–499)	159 (21–555)	141 *** (37–756)	244 (72–855)	0.01 ^b^
Total Tγδ cells	NC	73 (5.4–407)	91 (13–350)	92 (15–349)	122 (16–757)	NS

Results expressed as median (range) values. *, **, and *** indicate *p*-values ≤ 0.05, ≤0.01, and ≤0.001 for the comparison of variables from CLL cases studied at diagnosis vs. sex- and age-matched healthy donors. *p*-values refer to comparisons of: ^a^ stable vs. increasing CLL clones assessed at diagnosis; ^b^ stable vs. increasing CLL clones assessed at the last follow-up; ^c^ diagnosis vs. the last follow-up among cases with stable CLL clones; ^d^ diagnosis vs. the last follow-up among cases with increasing CLL clones. Abbreviations (alphabetical order): CLL, chronic lymphocytic leukemia; HD, healthy donors; MBC, memory B-cells; NS, no statistically significant differences found (*p*-value > 0.05); PC, plasma cells; sIg, surface immunoglobulin.

**Table 2:** Results of the univariate and multivariate analyses of those variables significantly associated with the kinetics of the clonal B-cell population in CLL stage A/0 patients.

	Univariate Analysis (Log-Rank Test)		Multivariate Analysis (Binary Logistic Regression)		Cox Regression	
	HR (95% CI)	*p*-Value	HR (95% CI)	*p*-Value	HR (95% CI)	*p*-Value
N. of clonal B-cells (≥15 × 10^9^ cells/L)	4.6 (2.0–10)	**≤0.001**	15 (1.1–222)	**0.046**	3.4 (1.1–10)	**0.03**
IgM^−^/IgM^+^ MBC ratio (≥1.7)	3.1 (1.3–7.2)	**0.013**	14 (0.64–291)	0.09	1.7 (0.73–5.0)	NS
N. of plasma cells (≥2.1 cells/µL)	1.9 (0.88–4.3)	0.07	3.6 (0.25–51)	NS	2.1 (0.73–6.1)	NS
N. of Tαβ CD4^+^CD8^lo^ T-cells (≤35 cells/µL)	3.1 (1.4–6.7)	**0.004**	33 (2.0–567)	**0.015**	3.3 (1.2–9.7)	**0.027**
*IGHV* mutational status (unmutated)	4.0 (1.2–13)	**≤0.001**	40 (2.3–686)	**0.011**	4.4 (1.7–11)	**0.002**

Only those continuous variables showing statistical significance or statistical significance trend (*p*-value ≤ 0.1) in the univariate analysis were considered in the multivariate analysis (after Boolean categorization). Abbreviations (alphabetical order): CLL, chronic lymphocytic leukemia; HR, hazard ratio; Ig, immunoglobulin; MBC, memory B-cells; N., number; NS, not statistically significant difference detected (*p*-value ≥ 0.1); 95% CI, 95% confidence interval.

## Data Availability

To protect participant privacy, the raw data are not publicly available. However, researchers may request access to anonymized data for further analysis.

## Supplementary Materials

The following supporting information can be downloaded at: https://www.mdpi.com/article/10.3390/cancers17030347/s1, Table S1: Combinations of fluorochrome-conjugated antibodies and phenotypic criteria used for the identification and characterization of peripheral blood (PB) B- and T-cell subsets by 8-color flow cytometry; Table S2: Panel of fluorochrome-conjugated probes used for interphase fluorescence in situ hybridization (iFISH) studies and the corresponding chromosomal regions targeted.; Table S3: Clinical and biological characteristics of the whole CLL cohort of patients included in the study at diagnosis vs. at the last follow-up time point; Table S4: Clinical and biological characteristics of CLL patients classified according to the kinetics of their blood-circulating B-cell clones at diagnosis vs. the last follow-up time-point; Table S5: Characterization of the *IGHV* sequence for each CLL patient included in the study according to the kinetics of the clonal B-cell population; Table S6: Soluble titers of CMV-specific IgM and IgG antibodies assessed in plasma of CLL stage A/0 patients studied at diagnosis; Table S7: Distribution of normal B- and T-cell populations in blood of the whole CLL cohort at baseline and at the last follow-up time point; Table S8: Distribution of B- and T-cell subsets in peripheral blood in healthy donors and CLL patients, stratified by age and the kinetics of the clonal B-cell populations (groups 40–59 years and 60–69 years); Table S9: Distribution of B- and T-cell subsets in peripheral blood in healthy donors and CLL patients, stratified by age and the kinetics of the clonal B-cell populations (groups 70–79 years and ≥80 years); Table S10: Univariate and multivariate analysis to determine the independent impact of the absolute number of B- and T-cell populations in the progression of the disease (time to first treatment); Figure S1: Gating strategy used for the flow cytometric identification of normal residual B- and T-cell populations in blood of CLL patients; Figure S2: Univariate analysis of prognostic factors associated with the time to first therapy in CLL.
