# Supplementary material for: Normal Residual Lymphoid Cell Populations in Blood as Surrogate Biomarker of the Leukemia Cell Kinetics in CLL BinetA/Rai 0
Source: Cancers (Basel). 2025 Jan 21;17(3):347. doi: 10.3390/cancers17030347 (PMC11815973; doi:10.3390/cancers17030347)
Supplement: Supplementary file 1 [file cancers-17-00347-s001.zip › cancers-3317590-supplementary.pdf]

SUPPLEMENTARY MATERIAL

SUPPLEMENTARY TABLES

TABLE S1. Combinations of fluorochrome-conjugated antibodies and phenotypic criteria used for the identification and characterization of peripheral blood (PB) B- and T-cell subsets by 8-color flow cytometry.

A. LST (EuroFlow® Lymphocyte Screening Tube) used for the identification of CLL cells:

**Fluorochrome**	**PacB**	**OC515**	**FITC**	**PE**	**PerCPCy5.5**	**PE-Cy7**	**APC**	**APC-H7**
**Marker**	CD20<br>CD4	CD45	CD8<br>anti-κ	CD56<br>anti-λ	CD5	CD19<br>TCRγδ	CD3	CD38
**Clone**	2H7<br>RPA-T4	HI30	UCHT4<br>polyclonal	C5.9<br>polyclonal	HIB19	HI10a<br>11F2	UCHT2	HB7
**Manufacturer**	eBioscience<br>BD	Cytognos	Cytognos	Cytognos	BD	BC<br>BD	BD	BD

B. Characterization tube for detailed analysis of normal residual B-cell subsets:

**Fluorochrome**	**PacB**	**OC515**	**FITC**	**PE**	**PerCPCy5.5**	**PE-Cy7**	**APC**	**APC-H7**
**Marker**	CD20	CD45	sIgM	CD10	CD5	CD19	CD27	CD38
**Clone**	2H7	HI30	G20-127	MEM-78	HIB19	HI10a	L128	HB7
**Manufacturer**	BD	Cytognos	BD	BD	BD	BC	BD	BD

C. Criteria used for the identification of the different populations of normal B-cells and PC in PB and their respective immunophenotypic profiles:

	**Peripheral blood B-cell population**				**Peripheral blood plasma cells**
Marker	Immature	Naive	Unswitched-memory	Switched-memory	
CD5	++	-/+	–	–	–
CD10	+	–	–	–	–
CD19	++	++	++	++	+
CD20	++	++	++	++	**– to +**
CD27	–	–	++	**– or +**	+++
CD38	++	–	–	–	+++
CD45	++	++	++	++	+
sIgM	++	+	+	–	**– or +^{dim}**

Data on the phenotypic profiles of different blood circulating normal B-cell and PC populations were defined according to previous criteria/ (references 31–34 of the main text and (1–4) of the Supplementary References).

Abbreviations (alphabetical order): **APC,** allophycocyanine; **APC-H7,** allophycocyanine-hilite®7; **FITC,** fluorescein isothiocyanate; **OC515,** orange Cytognos 515; **PacB**, pacific blue™; **PC,** plasma cells; **PE,** phycoerythrin; **PE-Cy7,** phycoerythrin-cyanine7; **PerCPCy5.5,** peridinin chlorophyll protein cyanine 5.5; **sIg,** surface immunoglobulin. Manufacturers (alphabetical order): Becton Dickinson Biosciences (BD), San José, CA; Beckman Coulter (BC), Brea, CA; Cytognos, Salamanca, Spain; Affymetrix eBioscience (eBioscience), San Diego, CA.

TABLE S2. Panel of fluorochrome-conjugated probes used for interphase fluorescence *in situ* hybridization (iFISH) studies and the corresponding chromosomal regions targeted.

Probe name	Fluorophore	Targeted chromosome band/region	Manufacturer
DLEU(13q14)/LAMP1(13q34)	Spectrum Aqua/	13q34 / 13q14.3	Werfen; Barcelona, Spain
CEP12	Spectrum Green	12p11.1-q11	Werfen; Barcelona, Spain
CEP11/ATM	Spectrum Green	11q22.3	Werfen; Barcelona, Spain
TP57/CEP17	Spectrum Orange	17p13	Cytocell; Cambridge, UK

TABLE S3. Clinical and biological characteristics of the whole CLL cohort of patients included in the study at diagnosis vs. at the last follow-up time point.

	Whole CLL cohort (n=69)		*p*-value
	Diagnosis	Last follow-up	
Sex (men/women)	32 / 37 (46% / 54%)		NA
Age (years)*	73 (49–87)	76 (52–88)	≤0.001
**Blood cell counts**			
Haemoglobin (g/L)*	140 (115–159)	134 (109–152)	≤0.001
N. of platelets ($\times 10^9$/L)*	191 (127–281)	172 (104–257)	≤0.001
N. of leukocytes ($\times 10^9$/L)*	20 (13–73)	21 (13–112)	≤0.001
N. of lymphocytes ($\times 10^9$/L)*	15 (9.3–69)	16 (8.4–102)	≤0.001
N. of clonal B cells ($\times 10^9$/L)*	11 (5.3–65)	13 (5.6–97)	≤0.001
**Biochemical parameters (serum)**			
B2-microglobulin (mg/L)*	2.4 (1.7–48)	2.7 (1.8–6.9)	≤0.001
LDH (U/L)*	340 (258–452)	338 (258–524)	NS
**% of cases with decreased serum Ig levels**			
IgG (<7 g/L)	5/69 (7.2%)	5/44 (11%)	NS
IgA (<0.7 g/L)	5/69 (7.2%)	5/44 (11%)	NS
IgM (<0.4 g/L)	21/69 (30%)	21/44 (48%)	NS
**N. and type of cytogenetic alterations**			
Del(13q14)(D13S25)	37/54 (69%)	NA	NA
Trisomy 12	7/56 (13%)	NA	NA
Del(11q)(*ATM*)	0/54 (0%)	NA	NA
Del(17p)(TP53)	3/55 (5.3%)	NA	NA
Presence of any alteration	41/56 (73%)	NA	NA
Single alteration/case	27/55 (49%)	NA	NA
>1 alteration/case	5/54 (6.7%)	NA	NA
***IGHV* mutational status**			
Unmutated (% UM)	12/62 (19%)	NA	NA
**Follow-up**			
Treatment-requiring progression	NA	14/69 (20%)	NA
Median time to therapy (months)	NA	Not reached	NA

Results expressed as number (percentage) of cases or as (*) median (range). P-values refer to comparisons of cases studied at diagnosis *vs.* the last follow-up time point. **Abbreviations (alphabetical order):** CLL, chronic lymphocytic leukemia; LDH, lactate dehydrogenase; N., number; NA, not applicable; NS, no statistically significant differences (*p*-value >0.05); PB, peripheral blood. UM, Unmutated *IGHV* sequence.

TABLE S4. Clinical and biological characteristics of CLL patients classified according to the kinetics of their blood-circulating B-cell clones at diagnosis vs. the last follow-up time-point.

	**Stable CLL clones (n=53)**		**Increasing CLL clones (n=16)**		***p*-value**
	**Diagnosis**	**Last Follow-up**	**Diagnosis**	**Last Follow-up**	
Sex (men/women)	25 / 28 (47% / 53%)		7 / 9 (44% / 56%)		NS
Age (years)*	73 (49–87)	76 (54–91)	72 (48–88)	73 (49–88)	0.001[c,d]
Blood cell counts					
Hemoglobin (g/L)*	141 (115–159)	136 (106–152)	133 (109–162)	128 (88–158)	≤0.005[c,d]
N. of platelets ($\times10^9$/L)*	194 (127–284)	180 (108–277)	175 (114–239)	138 (100–185)	≤0.02[b,c,d]
N. of leukocytes ($\times10^9$/L)*	19 (12–67)	19 (12–69)	46 (14–83)	71 (21–145)	≤0.001[a,b,d]
N. of lymphocytes ($\times10^9$/L)*	14 (8.7–50)	14 (7.5–65)	36 (7.2–82)	63 (18–131)	≤0.001[a,b,d]
N. of clonal B cells ($\times10^9$/L)*	9.3 (5.2–46)	10 (5.4–60)	32 (5.7–79)	61 (14–127)	≤0.001[a,b,d]
Biochemical parameters (serum)					
B2-microglobulin (mg/L)*	2.4 (1.7–4.7)	2.6 (1.8–6.1)	2.7 (1.2–5.6)	2.9 (1.8–14)	NS
LDH (U/L)*	338 (260–492)	344 (258–517)	342 (253–441)	328 (247–572)	NS
% of cases with decreased serum Ig levels					
IgG (<7 g/L)	4/53 (7.6%)	3/36 (8.3%)	1/16 (6.3%)	2/8 (25%)	NS
IgA (<0.7 g/L)	3/53 (5.7%)	3/36 (8.3%)	2/16 (13%)	2/8 (25%)	NS
IgM (<0.4 g/L)	14/53 (26%)	17/36 (47%)	7/16 (44%)	4/8 (50%)	NS
N. and type of cytogenetic alterations					
Del(13q14)(D13S25)	26/42 (62%)	NA	11/12 (92%)	NA	NS
Trisomy 12	7/44 (16%)	NA	0/12 (0%)	NA	NS
Del(11q)(*ATM*)	0/42 (0%)	NA	0/12 (0%)	NA	NS
Del(17p)(*TP53*)	3/42 (7.1%)	NA	0/13 (0%)	NA	NS
Presence of any alteration	31/43 (72%)	NA	10/13 (77%)	NA	NS
Single alteration/case	26/42 (62%)	NA	11/13 (85%)	NA	NS
>1 alteration/case	5/42 (12%)	NA	0/12 (0%)	NA	NS
IGHV mutational status					
Unmutated (%)	4/46 (8.7%)	NA	8/16 (50%)	NA	0.001
Follow-up					
Requirement for therapy	NA	6/53 (11%)	NA	8/16 (50%)	0.002
Median time to therapy (months)*	NA	Not reached	NA	51	0.001

Results expressed as number (percentage) of cases or (*) as median (range). [a] stable *vs.* increasing CLL clones assessed at diagnosis; [b] stable *vs.* increasing CLL clones assessed at the last follow-up; [c] diagnosis vs. the last follow-up among cases with stable CLL clones; [d] diagnosis *vs.* the last follow-up among cases with increasing CLL clones. **Abbreviations (alphabetical order):** CLL, chronic lymphocytic leukemia; LDH, lactate dehydrogenase; N., number; NA, not applicable; NS, no statistically significant differences detected (*p*-value >0.05); PB, peripheral blood.

TABLE S5 Characterization of the *IGHV* sequence for each CLL patient included in the study according to the kinetics of the clonal B-cell population.

Patient ID	CLL clone kinetics	Mut. Status	% of identity vs. germline	*IGHV* family usage	Patient ID	CLL clone kinetics	Mut. Status	% of identity vs. germline	*IGHV* family usage
CLLK002	Stable	M	93.72	*IGHV3-23*	CLLK038	Stable	M	86.76	*IGHV3-11*
CLLK004	Stable	M	85.67	*IGHV3-9*	CLLK040	Stable	M	88.54	*IGHV3-36*
CLLK005	Stable	M	88.66	*IGHV3-64*	CLLK041	Stable	M	95.04	*IGHV3-48*
CLLK006	Stable	UM	99.87	*IGHV1-69*	CLLK042	Stable	M	96.7	*IGHV2-5*
CLLK007	Stable	M	88.64	*IGHV4-30*	CLLK043	Stable	M	86.99	*IGHV3-48*
CLLK008	Stable	UM	98.75	*IGHV1-2*	CLLK045	Stable	M	95.3	*IGHV3-30*
CLLK009	Stable	M	91.4	*IGHV1-2*	CLLK046	Stable	M	91.85	*IGHV4-34*
CLLK010	Dynamic	UM	98.35	*IGHV3-30*	CLLK047	Stable	M	90.86	*IGHV3-10*
CLLK012	Dynamic	UM	100	*IGHV3-21*	CLLK050	Stable	M	83.68	*IGHV3-9*
CLLK013	Stable	M	84.03	*IGHV3-23*	CLLK052	Stable	M	94.12	*IGHV1-8*
CLLK014	Stable	M	92.9	*IGHV4-4*	CLLK053	Stable	M	94.47	*IGHV3-33*
CLLK015	Stable	M	92.82	*IGHV4-34*	CLLK054	Stable	M	79.17	*IGHV3-74*
CLLK016	Stable	M	92.1	*IGHV1-2*	CLLK055	Stable	M	92.82	*IGHV4-34*
CLLK017	Stable	UM	98.78	*IGHV4-34*	CLLK056	Stable	M	92.02	*IGHV4-34*
CLLK018	Stable	M	86.46	*IGHV3-74*	CLLK057	Dynamic	M	95.08	*IGHV1-2*
CLLK020	Stable	UM	100	*IGHV2-70*	CLLK058	Stable	M	97.59	*IGHV3-21*
CLLK021	Stable	M	94.94	*IGHV4-34*	CLLK059	Dynamic	UM	100	*IGHV4-34*
CLLK022	Stable	M	93.15	*IGHV1-8*	CLLK060	Dynamic	UM	99.35	*IGHV1-8*
CLLK023	Dynamic	M	86.32	*IGHV4-30*	CLLK061	Dynamic	UM	98.3	*IGHV3-23*
CLLK024	Stable	M	90.72	*IGHV3-23*	CLLK062	Dynamic	UM	99.1	*IGHV3-13*
CLLK025	Stable	M	90.36	*IGHV3-74*	CLLK063	Stable	M	88.8	*IGHV3-7*
CLLK026	Stable	M	89.95	*IGHV3-74*	CLLK065	Dynamic	UM	100	*IGHV2-70*
CLLK027	Dynamic	M	91.14	*IGHV4-59*	CLLK066	Stable	M	91.91	*IGHV4-34*
CLLK028	Stable	M	91.1	*IGHV2-5*	CLLK067	Dynamic	UM	99.6	*IGHV4-4*
CLLK029	Dynamic	M	88.8	*IGHV3-7*	CLLK068	Dynamic	M	95.52	*IGHV3-33*
CLLK030	Stable	M	87.61	*IGHV3-30*	CLLK069	Stable	M	86.11	*IGHV1-2*
CLLK031	Stable	M	91.2	*IGHV3-23*	CLLK070	Dynamic	M	85.43	*IGHV4-59*
CLLK032	Stable	M	85.45	*IGHV3-21*	CLLK071	Dynamic	M	81.6	*IGHV3-23*
CLLK033	Stable	M	89.07	*IGHV3-66*	CLLK072	Dynamic	M	92.3	*IGHV1-2*
CLLK034	Stable	M	97.26	*IGHV1-69*	CLLK073	Stable	M	96.34	*IGHV1-8*
CLLK035	Stable	M	78.47	*IGHV1-46*	CLLK074	Stable	M	91.37	*IGHV4-34*
CLLK037	Stable	M	67.35	*IGHV2-5*					

Abbreviations (alphabetical order): CLL, chronic lymphocytic leukemia; M, mutated; UM, unmutated.

TABLE S6. Soluble titers of CMV-specific IgM and IgG antibodies assessed in plasma of CLL stage A/0 patients studied at diagnosis

	**Stable**	**Increasing**	***p*-value**
% of positive IgM	0%	0%	NS
CMV-specific IgM titers (AU/mL)*	NA	NA	NS
% of positive IgG	100%	100%	NS
CMV-specific IgG titers (AU/mL)*	139 (33–243)	80 (9.5–221)	NS

Results expressed as number (percentage) of cases or (*) as median (range). **Abbreviations (alphabetical order): AU,** arbitrary units; CMV, cytomegalovirus; NA, not applicable; NS, no statistically significant differences detected (*p*-value >0.05).

TABLE S7. Distribution of normal B- and T-cell populations in blood of the whole CLL cohort at baseline and at the last follow-up time point.

	Sex- and age-matched HD	Whole CLL cohort (n=69) Diagnosis	Last follow-up	*p*-value
**N. of normal B cells/μL**				
Total B cells	160 (35–384)	82 *** (28–223)	62 (19–224)	0.009
Immature	5.7 (<0.01–36)	2.3 *** (0.24–17)	1.9 (<0.01–12)	NS
Naïve	79 (6.0–378)	12 *** (1.1–64)	8.8 (1.6–44)	0.05
MBC	51 (8.0–185)	64 (17–205)	37 (11–99)	0.001
MBC/Naïve ratio	0.67 (0.04–4.5)	3.9 *** (0.74–38)	3.1 (0.51–37)	NS
$sIgM^+$ MBC	26 (2.1–114)	10 *** (0.08–66)	11 (0.91–35)	NS
$sIgM^-$ MBC	29 (5.6–98)	50 *** (9.4–149)	21 (4.2–79)	NS
$sIgM^-/sIgM^+$ MBC ratio	1.2 (0.30–6.5)	2.3 *** (0.49–40)	1.9 (0.39–13)	NS
PC	1.8 (<0.01–24)	1.8 (<0.01–13)	2.8 (<0.01–31)	NS
**N. of normal T cells/μL**				
Total T-cells	1,129 (435–3,068)	2461 *** (1241–6549)	2532 (1365–5814)	NS
Tαβ $CD4^+CD8^-$ cells	620 (196–3,224)	1242 *** (640–3193)	1419 (525–2982)	NS
Tαβ $CD4^-CD8^+$ cells	366 (13-1,939)	753 *** (273–2474)	946 (267–2356)	NS
Tαβ $CD4^+CD8^-/CD4^-CD8^+$ cell ratio	1.9 (0.31–43)	1.7 (0.55–4.2)	1.6 (0.44–4.3)	NS
Tαβ $CD4^+CD8^+$ cells	7.4 (0.70–94)	48 *** (6.8–203)	37 (2.0–139)	NS
Tαβ $CD4^+CD8^{lo}$ cells	15 (2.4–436)	40 *** (6.2–556)	40 (6.8–303)	NS
Tαβ $CD4^-CD8^-$ cells	11 (2.0–39)	143 *** (31–499)	167 (37–636)	NS
Total Tγδ cells	NC	81 (6.3–405)	97 (13–484)	NS

Results expressed as median (range) values; * and *** ≤0.05 and ≤0.001 for the comparison of CLL cases at diagnosis vs. sex- and age-matched HD, respectively. *P*-values refer to the comparison of cases assessed at diagnosis *vs.* the last follow-up time point. **Abbreviations (alphabetical order):** CLL, chronic lymphocytic leukemia; HD, healthy donors; MBC, memory B cells; NC, not calculable; NS, no statistically significant differences found (*p*-value >0.05); PC, plasma cells; TCR, T-cell receptor.

TABLE S8. Distribution of B- and T-cell subsets in peripheral blood in Healthy Donors and CLL patients, stratified by age and the kinetics of the clonal B-cell populations (groups 40–59y and 60–69y).

	40–59y			69–69y			
	**HDs (n=50)**	**Stable CLL clones (n=9)**	**Increasing CLL clones (n=3)**	**HDs (n=65)**	**Stable CLL clones (n=12)**	**Increasing CLL clones (n=4)**	***p*-value**
N. of normal B cells/μL							
Total B cells	155 (41–299)	100 (73–223)	83 (75–194)	146 (54–324)	91 (15–430)	74 (67–138)	NS
Immature	6.9 (0.84–23)	1.8 (0.24–17)	2.6 (1.1–4.0)	4.8 (1.1–27)	2.0 (0.26–20)	1.9 (1.3–6.7)	NS
Naive	96 (26–244)	6.2 (1.1–64)	25 (21–29)	62 (29–176)	11 (0.53–64)	13 (7.5–15)	≤0.03[a,b,c,d]
MBC	32 (12–95)	37 (0.54–103)	48 (12–84)	28 (7.4–69)	6.7 (≤0.01–39)	8.3 (2.7–16)	NS
MBC/Naive ratio	32 (13–68)	67 (38–102)	57 (41–74)	24 (8.9–56)	58 (2.0–394)	50 (34–108)	≤0.002[a,b,d]
sIgM^{+} MBC	89 (32–157)	79 (64–205)	105 (52–158)	56 (18–110)	75 (5.7–403)	61 (45–111)	≤0.01[c,d]
sIgM^{-} MBC	0.76 (0.29–4.2)	8.3 (1.2–31)	3.3 (1.6–5.1)	0.74 (0.26–1.3)	6.3 (1.1–42)	3.3 (2.1–7.1)	≤0.02[a,b,d]
sIgM^{-}/sIgM^{+} MBC ratio	1.1 (0.30–2.4)	0.98 (0.64–40)	1.3 (0.74–1.8)	0.97 (0.48–2.6)	8.4 (0.46–101)	5.3 (2.1–9.6)	≤0.05[b,d]
PC	2.6 (0.33–24)	1.3 (0.01–12)	0.88 (0.01–3.4)	1.3 (0.40–7.3)	2.9 (0.01–8.5)	6.1 (1.1–13)	NS
N. of normal T cells/μL							
Total T-cells	988 (517–1934)	2779 (1422–4062)	1819 (1241–1880)	1301 (681–2098)	2863 (1400–10,154)	2756 (1472–7382)	≤0.006[a,b,d]
Tαβ CD4^{+}CD8^{-} cells	608 (226–1536)	1397 (886–2004)	1127 (687–1237)	753 (358–1309)	1695 (612–8303)	1179 (854–4140)	≤0.03[a,b,d]
Tαβ CD4^{-}CD8^{+} cells	315 (185–621)	1049 (248–1642)	470 (449–517)	423 (145–760)	673 (279–2387)	1375 (526–2474)	≤0.007[a,d]
Tαβ CD4^{+}CD8^{-}/ CD4^{-}CD8^{+} cell ratio	1.9 (0.76–4.1)	1.6 (0.55–4.2)	2.2 (1.5–2.6)	1.8 (0.65–3.5)	2.4 (0.26–8.9)	1.5 (0.60–1.7)	NS
Tαβ CD4^{+}CD8^{lo} cells	11 (1.1–65)	83 (6.2–767)	22 (21–34)	16 (1.2–93)	72 (13–562)	31 (9.1–165)	≤0.001[a,b]
Tαβ CD4^{-}CD8^{-} cells	38 (25–118)	109 (37–656)	125 (53–138)	39 (9.2–109)	215 (26–499)	131 (37–389)	≤0.04[a,b,c]

Results expressed as number (percentage) of cases or (*) as median (range). [a] HD *vs.* Stable CLL clones among age group 40-59y, [b] HD *vs.* Stable CLL clones among age group 60-69y, [c] HD *vs.* Increasing CLL clones among age group 40-59y, [d] HD *vs.* Increasing CLL clones among age group 60-69y. **Abbreviations (alphabetical order):** CLL, chronic lymphocytic leukemia; HD, healthy donors; NS, no statistically significant differences detected (*p*-value >0.05); MBC, memory B cells; PC, plasma cells.

TABLE S9. Distribution of B- and T-cell subsets in peripheral blood in Healthy Donors and CLL patients, stratified by age and the kinetics of the clonal B-cell populations (groups 70–79y and ≥80y).

		**69–69y**			**≥80y**		
	**HDs (n=86)**	**Stable CLL clones (n=14)**	**Increasing CLL clones (n=4)**	**HDs (n=45)**	**Stable CLL clones (n=18)**	**Increasing CLL clones (n=5)**	***p*-value**
N. of normal B cells/μL							
Total B cells	133 (44–298)	83 (22–229)	88 (74–148)	96 (35–255)	69 (28–224)	74 (18–95)	0.02[b]
Immature	6.7 (0.47–27)	3.5 (0.51–17)	1.4 (0.99–3.7)	4.7 (≤0.01–23)	2.3 (≤0.01–10)	1.7 (0.5–8.7)	NS
Naive	111 (18–283)	15 (3–109)	8.8 (4.8–15)	62 (15–193)	16 (1.5–35)	5.5 (2.3–9.6)	≤0.02[a,b,c]
MBC	21 (7.6–35)	14 (3.6–66)	2.6 (1.3–16)	13 (5.3–57)	10 (≤0.01–29)	4.2 (1.2–19)	NS
MBC/Naive ratio	29 (6.3–98)	43 (9.4–117)	72 (62–102)	22 (7.1–84)	35 (12–218)	48 (3.0 –74)	≤0.003[a,b,c,d]
$sIgM^{+}$ MBC	53 (18–125)	58 (17–140)	75 (63–118)	36 (16–119)	45 (17–218)	59 (4.2–80)	≤0.04[c,d]
$sIgM^{-}$ MBC	0.51 (0.15–2.7)	3.6 (0.74–11)	5.6 (3.5–7.6)	0.90 (0.21–3.5)	2.3 (≤0.01–65)	5.4 (1.8–16)	0.005[c]
$sIgM^{-}/sIgM^{+}$ MBC ratio	1.4 (0.53–4.6)	1.6 (0.30–4.4)	17 (4.0–18)	1.3 (0.76–4.1)	1.6 (0.75–208)	5.1 (1.4–13)	≤0.04[c,d]
PC	1.8 (0.33–22)	1.85 (0.01–33)	2.9 (2.2–17)	1.8 (0.18–17)	1.5 (0.01–6.4)	6.1 (0.01–19)	NS
N. of normal T cells/μL							
Total T-cells	998 (565–2315)	2453 (1248–6763)	2178 (1890–2461)	1020 (690–2293)	2518 (984–6549)	1890 (1170–5970)	≤0.005[a,b,c]
Tαβ $CD4^{+}CD8^{-}$ cells	580 (294–1219)	1144 (683–3891)	1153 (943–1760)	602 (301–1567)	1224 (532–3193)	1009 (648–3129)	≤0.03[a,b,c,d]
Tαβ $CD4^{-}CD8^{+}$ cells	329 (152–990)	652 (273–2053)	608 (502–647)	426 (139–1337)	1027 (178–3130)	654 (194–1842)	≤0.02[a,b,c]
Tαβ $CD4^{+}CD8^{-}$/ $CD4^{-}CD8^{+}$ cell ratio	1.9 (0.78–4.2)	1.8 (0.94–4.1)	1.8 (1.6–3.5)	1.6 (0.50–4.4)	1.4 (0.20–3.7)	2.1 (1.5–3.4)	NS
Tαβ $CD4^{+}CD8^{lo}$ cells	12 (2.0–56)	45 (12–198)	12 (5.4–29)	17 (5.3–224)	41 (5.6–556)	74 (13–199)	0.001[a]
Tαβ $CD4^{-}CD8^{-}$ cells	39 (9.1–167)	161 (27–576)	224 (130–461)	27 (6.6–133)	134 (31–384)	165 (114–756)	≤0.003[a,b,c,d]

Results expressed as number (percentage) of cases or (*) as median (range). [a] HD *vs.* Stable CLL clones among age group 40-59y, [b] HD *vs.* Stable CLL clones among age group 60-69y, [c] HD *vs.* Increasing CLL clones among age group 40-59y, [d] HD *vs.* Increasing CLL clones among age group 60-69y. **Abbreviations (alphabetical order):** CLL, chronic lymphocytic leukemia; HD, healthy donors; NS, no statistically significant differences detected (*p*-value >0.05); MBC, memory B cells; PC, plasma cells.

TABLE S10. Univariate and multivariate analyses to determine the independent impact of the absolute number of B- and T-cell populations in the progression of the disease (time to first treatment).

	**Univariate analysis (log-rank test)**		**Multivariate analysis (Binary logistic regression)**		**Cox regression**	
	HR (95% CI)	*p*-value	HR (95% CI)	*p*-value	HR (95% CI)	*p*-value
N. of clonal B cells ($\geq 15\times10^9$ cells/L)	5.0 (1.7–15)	**0.003**	11 (0.80–148)	**0.07**	7.2 (1.9–28)	**0.004**
IGHV mutational status (unmutated)	54 (11–261)	**<0.001**	42 (1.8–964)	**0.02**	17 (1.0–296)	**0.05**
N. of plasma cells (≥3.1 cells/μL)	7.2 (2.3–23)	**0.007**	9.8 (0.79–123)	NS	0.68 (0.13–3.6)	NS
N. of $sIgM^-$ MBCs (≥50 cells/μL)	2.1 (068–6.1)	NS	0.82 (0.12–5.7)	NS	2.6 (1.6–24)	NS
del17p(*TP53*) (altered)	39 (1.9–776)	**0.02**	51 (0.85–3111)	NS	17 (0.98–295)	NS

Only those continuous variables showing statistical significance or statistical significance trend (*p*-value ≤0.1) in the univariate analysis were considered in the multivariate analysis (after Boolean categorization). **Abbreviations (alphabetical order):** CLL, chronic lymphocytic leukemia; HR, hazard ratio; Ig, immunoglobulin; MBC, memory B cells; N., number; NS, not statistically significant difference detected (*p*-value ≥0.1); 95% CI, 95% confidence interval.

SUPPLEMENTARY FIGURES

FIGURE S1. Gating strategy used for the flow cytometric identification of normal residual B- and T-cell populations in blood of CLL patients. Abbreviations (alphabetical order): **APC,** allophycocyanine; **APC-H7,** allophycocyanine-hilite®7; **FITC,** fluorescein isothiocyanate; **MBC,** memory B cells; **OC515,** orange Cytognos 515; **PacB,** pacific blue™; **PE,** phycoerythrin; **PE/Cy7,** phycoerythrin/cyanine7; **PerCPCy5.5,** peridinin chlorophyll protein cyanine 5.5

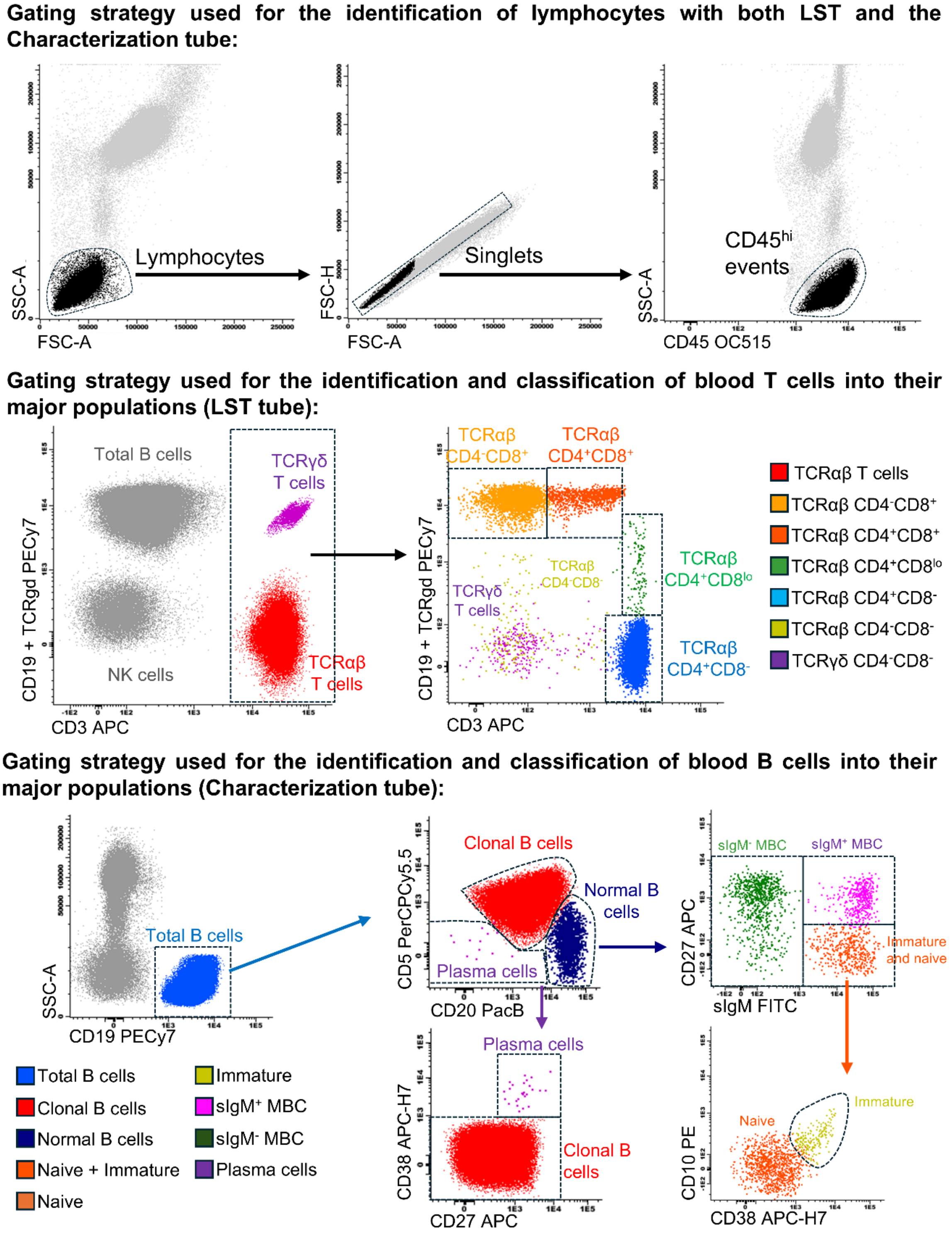

FIGURE S2. Univariate analysis of prognostic factors associated with the time to first therapy in CLL.

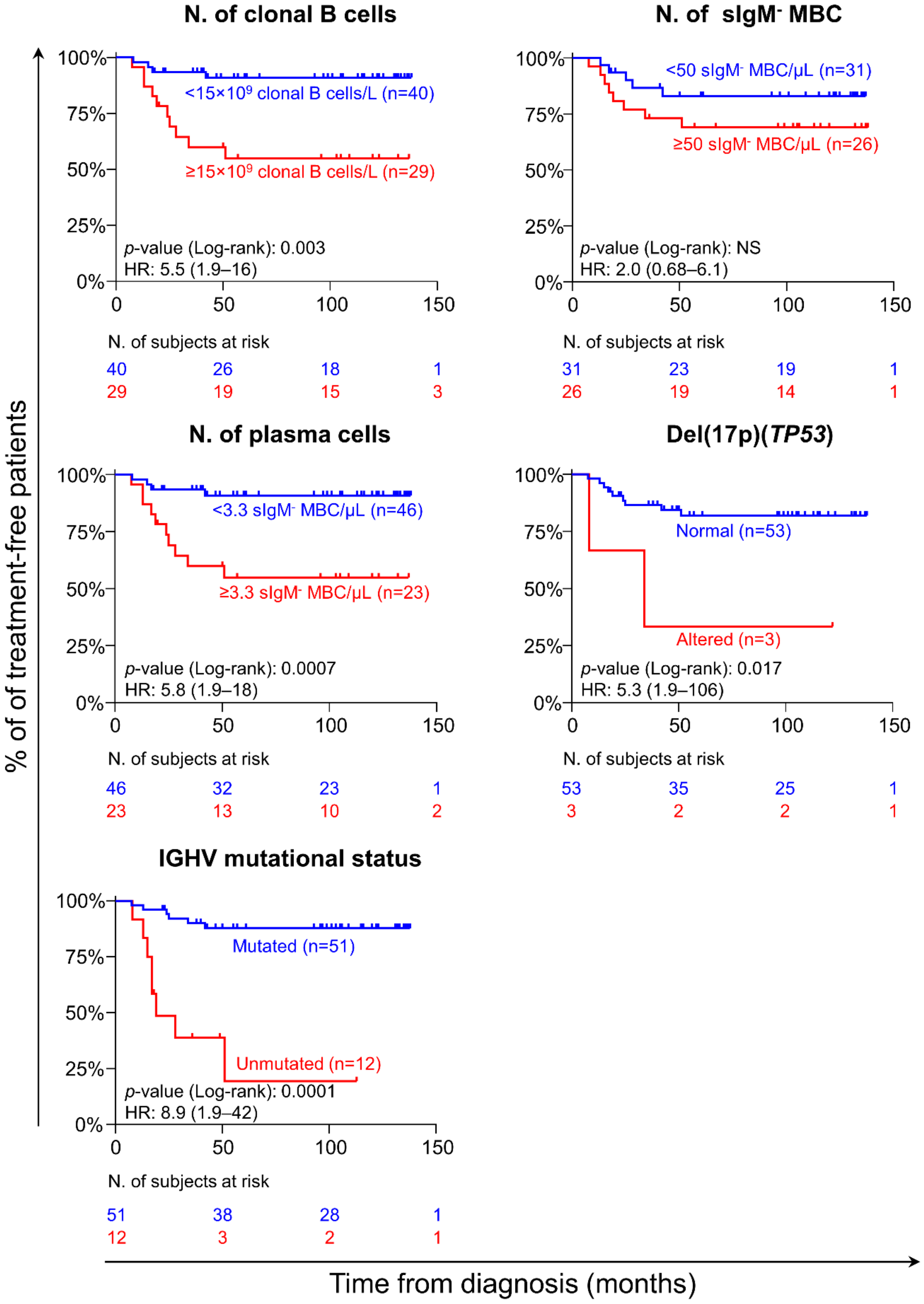

Abbreviations (alphabetical order): *IGHV*, immunoglobulin heavy chain gene; MBC, memory B cells; N., number.

SUPPLEMENTARY REFERENCES:

1. Perez-Andres M, Paiva B, Nieto WG, Caraux A, Schmitz A, Almeida J, Vogt RF, Marti GE, Rawstron AC, Van Zelm MC, et al. Human peripheral blood B-cell compartments: a crossroad in B-cell traffic. *Cytometry B Clin Cytom* (2010) 78 Suppl 1:S47-60. doi: 10.1002/cyto.b.20547

2. Blanco E, Pérez-Andrés M, Arriba-Méndez S, Contreras-Sanfeliciano T, Criado I, Pelak O, Serra-Caetano A, Romero A, Puig N, Remesal A, et al. Age-associated distribution of normal B-cell and plasma cell subsets in peripheral blood. *J Allergy Clin Immunol* (2018) 141:2208-2219.e16. doi: 10.1016/j.jaci.2018.02.017

3. Blanco E, Perez-Andres M, Sanoja-Flores L, Wentink M, Pelak O, Martín-Ayuso M, Grigore G, Torres-Canizales J, López-Granados E, Kalina T, et al. Selection and validation of antibody clones against IgG and IgA subclasses in switched memory B-cells and plasma cells. *J Immunol Methods* (2019) 475:112372. doi: 10.1016/j.jim.2017.09.008

4. Hultin LE, Chow M, Jamieson BD, O'Gorman MRG, Menendez FA, Borowski L, Denny TN, Margolick JB. Comparison of interlaboratory variation in absolute T-cell counts by single-platform and optimized dual-platform methods. *Cytometry B Clin Cytom* (2010) 78:194–200. doi: 10.1002/cyto.b.20500
